# Progress Toward Poliovirus Containment Implementation — Worldwide, 2019–2020

**DOI:** 10.15585/mmwr.mm6937a7

**Published:** 2020-09-18

**Authors:** Daphne B. Moffett, Anna Llewellyn, Harpal Singh, Eugene Saxentoff, Jeffrey Partridge, Liliane Boualam, Mark Pallansch, Steven Wassilak, Humayun Asghar, Sigrun Roesel, Varja Grabovac, Gloria Rey-Benito, Jacob Barnor, Andros Theo, Joseph Swan, Maria Iakovenko, Najam Baig, Santosh Gurung, Ekkehart Pandel, Michel Zaffran

**Affiliations:** ^1^World Health Organization, Geneva, Switzerland; ^2^Global Polio Eradication Initiative Containment Management Group, Geneva, Switzerland; ^3^CDC; ^4^Bill and Melinda Gates Foundation, Seattle, Washington; ^5^Rotary International, Evanston, Illinois.

Since 1988, when World Health Organization (WHO) Member States and partners launched the Global Polio Eradication Initiative, the number of wild poliovirus (WPV) cases has declined from 350,000 in 125 countries to 176 in only two countries in 2019 (*1*). The Global Commission for the Certification of Poliomyelitis Eradication (GCC) declared two of the three WPV types, type 2 (WPV2) and type 3 (WPV3), eradicated globally in 2015 and 2019, respectively (*1*). Wild poliovirus type 1 (WPV1) remains endemic in Afghanistan and Pakistan (*1*). Containment under strict biorisk management measures is vital to prevent reintroduction of eradicated polioviruses into communities from poliovirus facilities. In 2015, Member States committed to contain type 2 polioviruses (PV2) in poliovirus-essential facilities (PEFs) certified in accordance with a global standard (*2*). Member states agreed to report national PV2 inventories annually, destroy unneeded PV2 materials, and, if retaining PV2 materials, establish national authorities for containment (NACs) and a PEF auditing process. Since declaration of WPV3 eradication in October 2019, these activities are also required with WPV3 materials. Despite challenges faced during 2019–2020, including the coronavirus disease 2019 (COVID-19) pandemic, the global poliovirus containment program continues to work toward important milestones. To maintain progress, all WHO Member States are urged to adhere to the agreed containment resolutions, including officially establishing legally empowered NACs and submission of PEF Certificates of Participation.

## Background

The Global Polio Eradication Initiative has achieved its progress through extensive use of trivalent oral poliovirus vaccine (tOPV, which consists of live, attenuated Sabin vaccine strain types 1, 2, and 3). Despite the substantial advancement toward eradication attained using this vaccine, in areas with low population immunity, prolonged transmission of Sabin vaccine virus can lead to viral mutations that result in development of neurovirulent vaccine-derived polioviruses (VDPVs) (*1*). Outbreaks can result from VDPVs that are transmitted in a community and are known as circulating VDPVs (cVDPVs). Since the majority of cVDPV outbreaks were caused by the type 2 oral poliovirus vaccine strain (OPV2), a coordinated global switch in vaccines was conducted in 2016, replacing the use of tOPV with bivalent OPV (bOPV, which consists of Sabin strain types 1 and 3) (*3*). PV2 disease immunity in the community was to be provided by high coverage with tOPV before the switch as well as a recommended single dose of injectable inactivated polio vaccine (IPV) to help protect against paralysis; however, suboptimal vaccination coverage and IPV manufacturing shortages have led to substantial PV2 immunity gaps in many countries (*4*). Since the switch, many countries, particularly in Africa, have experienced cVDPV2 outbreaks (*1*). To combat these outbreaks, vaccination responses with monovalent OPV2 (mOPV2) have been implemented in approximately two dozen countries. However, waning type 2 immunity and delayed and low-quality outbreak responses have resulted in spread of existing outbreaks and emergence of new cVDPV2 outbreaks, leading to a significant increase in areas affected by cVDPV2 across parts of Africa and Asia (*5*). A novel OPV type 2, (nOPV2) engineered to be more genetically stable to prevent seeding of cVDPV2 outbreaks, is expected to be available for initial use in cVDPV2 outbreak response vaccination campaigns in October 2020 under WHO’s Emergency Use Listing (*5*).

## Global Poliovirus Containment Certification Status

In 2015, WHO Member States resolved to contain all PV2 viruses (i.e., wild, VDPV2, and OPV2/Sabin2) in designated PEFs certified by the WHO Global Action Plan to minimize poliovirus facility–associated risk after type-specific eradication of WPVs and sequential cessation of oral polio vaccine use (GAPIII) (*2*). As of August 2020, a total of 25 countries planned to retain PV2 materials in 73 designated PEFs (Figure). However, no facilities have yet been certified as GAPIII compliant. NACs have been established in 22 of these countries. Some countries, including China, Romania, and the United Kingdom have not yet delegated legal responsibility to their NACs. Of the 73 designated PEFs, 32 have been awarded GCC-endorsed Certificates of Participation (which validate successful enrollment in the WHO GAPIII-Containment Certification Scheme) (*6*). The deadline for PV2 PEFs to submit Certificate of Participation applications to NACs was December 31, 2019 (*7*). PV2 facilities and the respective NACs that have missed this deadline are urged to expeditiously submit these applications. The Certificates of Participation that have been awarded are due to expire in April 2021, by which time facilities were expected to have interim or full certificates of containment awarded after full GAPIII audits. Challenges in auditor qualification and delays related to COVID-19 might require revision of deadlines.

**FIGURE:**
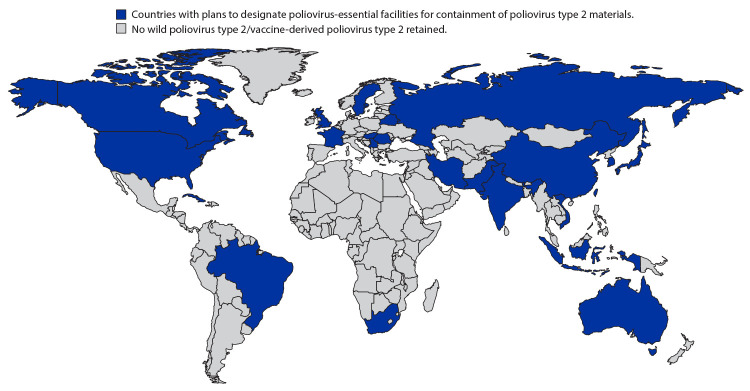
Twenty-five countries that currently plan to retain all type 2 polioviruses in 73 designated poliovirus-essential facilities

Although the first GAPIII certification audits were planned for 2020, the COVID-19 pandemic has delayed in-person audit activities. Qualification of 10 GAPIII-Containment Certification Scheme lead auditors, anticipated by the end of 2020, has been postponed because of challenges in creating a global auditor qualification program and disruptions caused by the global COVID-19 crisis. In response to current challenges, a revised multiyear plan, which includes the qualification of auditors and the certification of facilities, is currently being prepared. In 2019, the Global Polio Eradication Initiative facilitated four GAPIII PEF webinars, and six GAPIII in-depth weeklong in-person trainings were conducted worldwide to help prepare PEFs to implement strict GAPIII requirements. In addition, the WHO secretariat and members of the GCC Containment Working Group have engaged in multiple NAC network and bilateral meetings to expedite the certification process.

## Advisory Group Decisions

The Containment Advisory Group (CAG) was established in 2017 to advise the WHO Director-General regarding technical considerations for the implementation of GAPIII. In July 2019, CAG discussed revision of GAPIII,* which has undergone major and minor revisions since it was written in 2015, including 1) the replacement of Annex 4 of GAPIII with the GAPIII-Containment Certification Scheme^†^ and 2) a shift from OPV/Sabin potentially infectious materials being subject to GAPIII PEF containment requirements to other guidance requirements.^§^ WHO anticipates publishing an updated document in 2021 that will include all relevant revisions. In its March 2020 meeting, CAG agreed that, although nOPV types 1 and 3 contain a modified type-2 nonstructural region, nOPV1 and nOPV3 should be considered as PV type 1 or 3 for purposes of containment.^¶^

## Evolving Use of Live Poliovirus Vaccines

Since the 2016 global switch from tOPV to bOPV, the WHO Global Polio Laboratory Network detected 41 cVDPV2 outbreaks, many of which were likely seeded by mOPV2 use in outbreak responses (*4*). nOPV2 is predicted to have a substantially lower risk of seeding cVDPV2 outbreaks compared with mOPV2. Based on all available safety data, CAG has granted nOPV2 a waiver to be manufactured and used in outbreak response outside GAPIII containment conditions. However, nOPV2 is subject to other containment and safety requirements including rigorous inventorying, vial tracking, and enhanced environmental surveillance in countries where it is deployed. Once Phase III clinical trial data are available, CAG will review together with surveillance data from outbreak response countries to monitor any need to modulate GAPIII containment in facilities handling nOPV2.

Because of ongoing challenges in control of WPV1 transmission in Afghanistan and Pakistan coupled with expanding cVDPV2 outbreaks, the Global Polio Eradication Initiative and country ministries of health have agreed to use tOPV for outbreak response in areas where more than one serotype is circulating (*8*). The return to tOPV use is anticipated to quickly raise population intestinal immunity against all three polio virus types and address the dual challenges of WPV1 and cVDPV2 transmissions in those countries.

Approval for release of tOPV for selected outbreak response in areas with cocirculation will be granted from the WHO director general if recommended by the mOPV2 Advisory Group (*8*). National EPI teams should report to their national containment authorities on the use and management of tOPV and nOPV2 vaccines. As is currently required for mOPV2, national containment authorities will also be required to report any tOPV and nOPV2 inventories and relevant materials to their respective polio eradication National Certification Committee each year.

## Discussion

In 2018, the 71st World Health Assembly resolution urged all Member States to accelerate poliovirus containment efforts. Since then, global progress toward poliovirus containment has continued despite challenges and delays. As with all global programs, the COVID-19 pandemic has disrupted some poliovirus containment activities, including planned GAPIII certification audits, a vital component of the program. WHO Member States with proposed PV2 PEFs are urged to reassess the necessity of retaining materials. Upon declaration of the eradication of WPV3 in 2019, WPV3/cVDPV3 materials became subject to the same containment requirements as those for PV2. Because there are no immediate plans to remove the type 3 vaccine strain from use, OPV3 is not currently subject to containment requirements.

Ongoing cVDPV2 outbreaks continue to complicate global polio outbreak responses and poliovirus containment activities. Once a cVDPV2 outbreak is closed in outbreak countries, repeat inventories of cVDPV2 materials and destruction or transfer to a PEF should be documented. In addition, for all OPV2 materials (including retained stool specimens), mOPV2, tOPV, and nOPV2 vials should be tracked from point of release to use or destruction. Even with current disruptions in other aspects of poliovirus containment, all WHO Member States with PEFs need to adhere to World Health Assembly resolutions, including officially establishing legally empowered NACs and submission of PEF Certificates of Participation.

**Summary**

**What is already known about this topic?**

Containment of poliovirus materials is essential to establishing and maintaining global poliovirus eradication.

**What is added by this report?**

Wild poliovirus type 2 was declared eradicated in 2015; 25 countries have designated 73 poliovirus-essential facilities to retain poliovirus type 2 materials. Wild poliovirus type 3 materials have been subject to containment requirements since the virus was declared eradicated in 2019. Just as with type 2 monovalent Sabin oral poliovirus vaccine (OPV), countries using novel type 2 OPV or trivalent Sabin OPV for outbreak response should track and report related materials according to poliovirus containment requirements.

**What are the implications for public health practice?**

Countries are urged to expedite vital poliovirus containment activities, globally agreed upon in 2015, that have been delayed.

## Footnotes

* http://polioeradication.org/wp-content/uploads/2020/04/Fourth-meeting-of-the-Containment-Advisory-Group-2019071516.pdf.

† Annex 4 of GAPIII stated that PEF certification audits would be performed by WHO. The GPIII-Containment Certification Scheme was later developed that shifted the responsibility to countries to ensure GAPIII audits were performed by auditors qualified per the requirements of the scheme.

§ http://polioeradication.org/wp-content/uploads/2018/06/polio-containment-guidance-for-non-poliovirus-facilities-20180614-en.pdf.

¶ http://polioeradication.org/wp-content/uploads/2020/04/CAG-TC-20-March-2020-NFR.pdf.
